# Identification of critical isthmus using coherent mapping in patients with scar‐related atrial tachycardia

**DOI:** 10.1111/jce.14457

**Published:** 2020-04-06

**Authors:** Jennifer Jeanne B. Vicera, Yenn‐Jiang Lin, Po‐Tseng Lee, Shih‐Lin Chang, Li‐Wei Lo, Yu‐Feng Hu, Fa‐Po Chung, Chin‐Yu Lin, Ting‐Yung Chang, Ta‐Chuan Tuan, Tze‐Fan Chao, Jo‐Nan Liao, Cheng‐I Wu, Chih‐Min Liu, Chung‐Hsing Lin, Chieh‐Mao Chuang, Chun‐Chao Chen, Chye Gen Chin, Shin‐Huei Liu, Wen‐Han Cheng, Le Phat Tai, Sung‐Hao Huang, Ching‐Yao Chou, Isaiah Lugtu, Ching‐Han Liu, Shih‐Ann Chen

**Affiliations:** ^1^ Division of Cardiology, Department of Medicine, Heart Rhythm Center Taipei Veterans General Hospital Taipei Taiwan; ^2^ Department of Medicine National Yang‐Ming University School of Medicine Taipei Taiwan

**Keywords:** activation mapping, atrial tachycardia, coherent mapping, focal atrial tachycardia, scar‐related macro re‐entrant

## Abstract

**Introduction:**

Accurate identification of slow conducting regions in patients with scar‐related atrial tachycardia (AT) is difficult using conventional electrogram annotation for cardiac electroanatomic mapping (EAM). Estimating delays between neighboring mapping sites is a potential option for activation map computation. We describe our initial experience with CARTO 3 Coherent Mapping (Biosense Webster Inc,) in the ablation of complex ATs.

**Methods:**

Twenty patients (58 ± 10 y/o, 15 males) with complex ATs were included. We created three‐dimensional EAMs using CARTO 3 system with CONFIDENSE and a high‐resolution mapping catheter (Biosense Webster Inc). Local activation time and coherent maps were used to aid in the identification of conduction isthmus (CI) and focal origin sites. System‐defined slow or nonconducting zones and CI, defined by concealed entrainment (postpacing interval < 20 ms), CV < 0.3 m/s and local fractionated electrograms were evaluated.

**Results:**

Twenty‐six complex ATs were mapped (mean: 1.3 ± 0.7 maps/pt; 4 focal, 22 isthmus‐dependent). Coherent mapping was better in identifying CI/breakout sites where ablation terminated the tachycardia (96.2% vs 69.2%; *P* = .010) and identified significantly more CI (mean/chamber 2.0 ± 1.1 vs 1.0 ± 0.7; *P* < .001) with narrower width (19.8 ± 10.5 vs 43.0 ± 23.9 mm; *P* < .001) than conventional mapping. Ablation at origin and CI sites was successful in 25 (96.2%) with long‐term recurrence in 25%.

**Conclusions:**

Coherent mapping with conduction velocity vectors derived from adjacent mapping sites significantly improved the identification of CI sites in scar‐related ATs with isthmus‐dependent re‐entry better than conventional mapping. It may be used in conjunction with conventional mapping strategies to facilitate recognition of slow conduction areas and critical sites that are important targets of ablation.

## 1 INTRODUCTION

Conventional mapping of complex scar‐related atrial tachycardias (ATs) can be challenging.^1^ Three‐dimensional (3D) electroanatomic mapping (EAM) systems facilitate such difficult interventional ablation procedures and allow accurate navigation to a predefined site with favorable spatial resolution and visualization of the activation sequence (activation mapping) and voltage information (voltage mapping).^2^ An important factor that influences the accuracy of the activation map includes the consistency of electrogram annotation which is dependent on numerous algorithms (eg, peak amplitude or rapid downstroke of the unipolar signal) that can be selected for automatic signal annotation.^3^ The presence of scarred tissue from prior surgery or ablation can cause a variable delay between the onset of the local electrogram and the time to the peak amplitude.^4^ This limits the accuracy of the activation mapping which relies on the correct annotation of the impulse timing different from that of a reference electrode. Other limitations of activation mapping include choosing the mapping window of interest based on an arbitrarily defined early and late activation and difficulty in differentiating active diastolic activity that is part of the re‐entrant circuit from passive diastolic activity recorded in scar unrelated to the tachycardia.^5^

An algorithm incorporating conduction velocity vectors computed taking into account a global best‐fit solution allows the management of such complex electrograms, identifies abnormal substrate with slow or nonconducting (SNO) zones and facilitates interpretation of such complex AT mechanisms.^5^ In this pilot study, we evaluated the usefulness of this novel algorithm of coherent maps with conduction velocity vectors in correctly identifying critical sites for the ablation of complex ATs in patients with scar‐related AT in comparison with standard activation maps.

## 2 METHODOLOGY

### 2.1 Study population

This is a prospective observational study of 20 consecutive patients undergoing radiofrequency catheter ablation of complex AT at the Heart Rhythm Center, Department of Medicine, Taipei Veterans General Hospital from January 2018 to September 2018. Complex AT was defined as: AT occurring after a previous cardiac procedure including catheter ablation, cardiac surgery for congenital, and/or valvular heart disease or other underlying heart diseases. Patients meeting one of the criteria for complex AT were included in the study. Exclusion criteria were: (a) age younger than 18 years, (b) reversible cause of arrhythmia, (c) presence of an intracardiac thrombus, (d) contraindications to oral or intravenous anticoagulants, (e) pregnancy, (f) irregular/unstable rhythm, and (g) nonsustained AT during mapping.^1^ The coherent map was applied and evaluated prospectively for mapping and guiding ablation in all 20 patients. All patients provided informed consent for the electrophysiologic study and ablation. This study was approved by the Institutional Review Board of the Taipei Veterans General Hospital (IRB Approval #2019‐02‐018A). All patients with suspected left atrial (LA) focus had either a transesophageal echocardiography or cardiac computed tomography scan done to exclude LA appendage thrombus within 2 days before procedure.

### 2.2 Electrophysiological study

The details of mapping have been described previously. In brief, each patient underwent an electrophysiological study and catheter ablation in the postabsorptive, nonsedated state,^6^, ^7^ except for one patient who underwent epicardial mapping under general anesthesia. Surface electrocardiography (ECG) and bipolar electrograms were continuously monitored with LabSystem Pro (Bard Electrophysiology, Lowell, MA), sampled at 1 kHz and bandpass filtered at 30 to 300 Hz. A multielectrode mapping catheter (Lasso Nav or PentaRay Nav; Biosense Webster Inc, Diamond Bar, CA) and a 3.5 mm open‐irrigated tip ablation catheter (ThermoCool; Biosense Webster Inc,) were placed through sheaths into the atrial chamber of interest.

Activation mapping was directly performed for all patients who were in incessant AT on presentation and AT induction was done for those in sinus rhythm before mapping. Induction of AT was done with stimulation from the proximal and distal coronary sinus.^8^, ^9^ If AT is not induced with programmed electrical stimulation, intravenous isoproterenol (at graded dosages from 1 to 4 μg/min) was infused until AT developed or the sinus rate increased to 20% above the resting value.^8^, ^10^, ^11^ After inducing sustained AT, EAM was performed and the suspected circuits were confirmed with entrainment maneuvers, and postpacing interval (PPI) analyses from multiple sites to identify the mechanism of the AT. Pacing sites with a PPI less than or equal to 20 ms of the CL were considered as part of the circuit. Macroreentrant AT accounted for at least 80% of the tachycardia CL on activation mapping.^12^ The absence of entrainment from multiple pacing sites and a centrifugal activation from a focal site indicate focal AT as the mechanism.^9^, ^11^, ^13^, ^14^ Transseptal puncture under right atriography guidance was performed to access the LA, if needed, and intravenous heparin was administered to maintain an activated clotting time between 250 and 350 seconds.

### 2.3 Electroanatomical mapping

#### 2.3.1 Data collection settings

**Figure 1:**
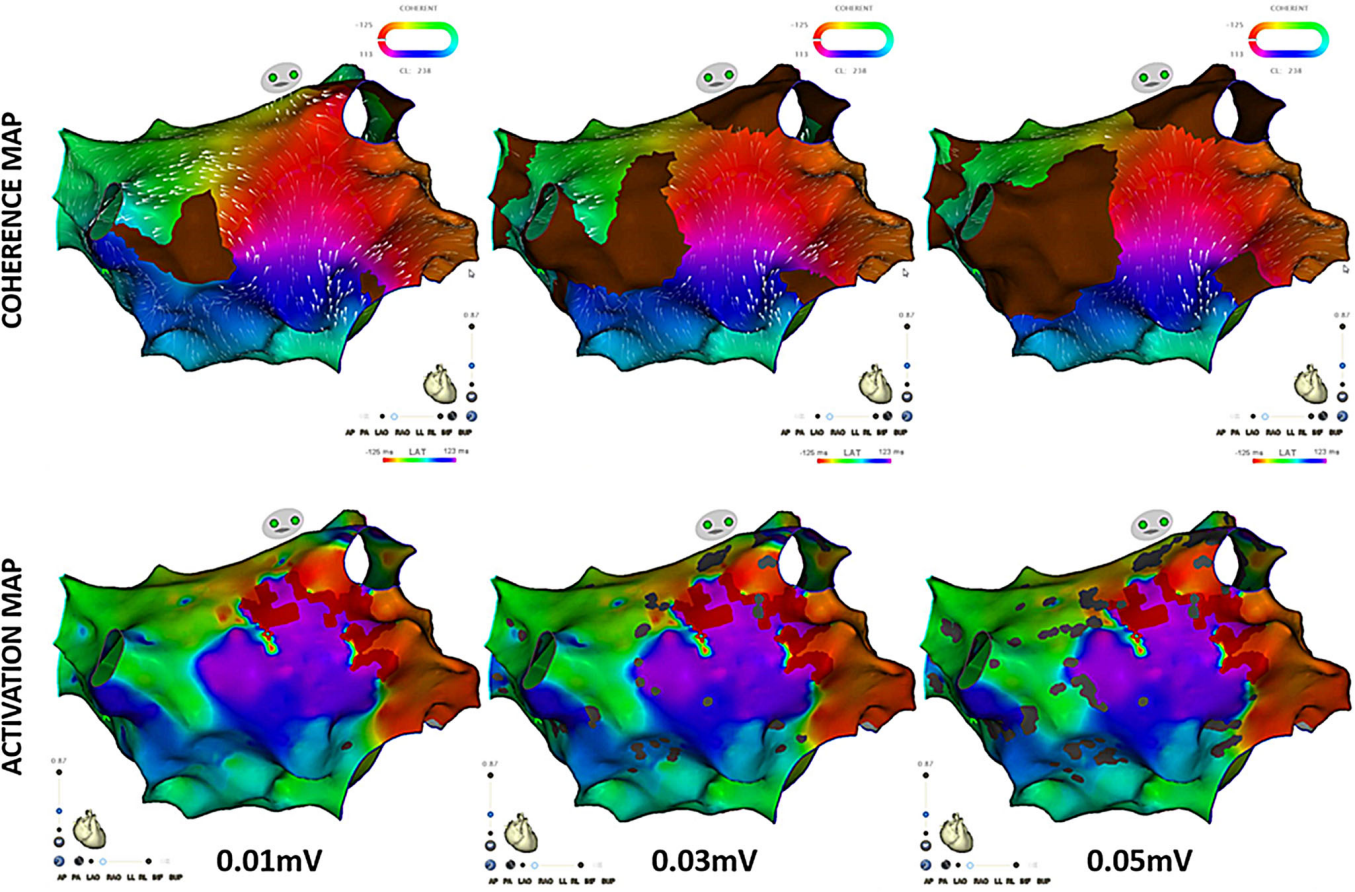
Effect of different scar settings on activation and coherent maps for identification of isthmus site top panels correspond to coherent maps. The bottom panels correspond to LAT maps. The scar threshold setting was adjusted with 0.01, 0.03, and 0.05 mV thresholds from left to right, respectively. The figure demonstrates increasing SNO areas on the recalculated coherent maps and increasing patches of gray areas on the LAT maps as the scar threshold setting is increased. The changes are more apparent on the coherent maps than the LAT maps. The 0.01 mV is shown only for demonstration purpose but was not applied during the actual cases because a threshold less than 0.03 mV is below the noise threshold for the CARTO system. LAT, local activation time; SNO, slow or nonconduction areas

All maps were acquired using a multielectrode mapping catheter (Lasso Nav or PentaRay Nav; Biosense Webster Inc,) and CARTO 3 system CONFIDENSE module with the continuous acquisition of electroanatomical (EA) points that meet a set of predefined filters. Points were accepted only if they pass all the selected filters including cycle length stability within a 5% range of the tachycardia cycle length, local activation time (LAT) stability within 4 ms and an electrode position stability within 4 mm. A tissue proximity filter based on catheter location and impedance measurements was used to determine electrode proximity to cardiac tissue. The greatest negative deflection (–dV/dt) in each distal unipolar signal was used for the LAT calculation. Map consistency display was used to search for, and filter points whose LAT values are not consistent with the LAT values of neighboring points to highlight these outliers. The interpolation threshold of the color fill‐in of the reconstruction was set at 5% during map acquisition to permit a relatively uniform density of mapping points.^15^ Bipolar electrograms were filtered between 16 and 500 Hz, unipolar electrograms between 2 and 240 Hz and recorded digitally.^7^ SNO zones were defined by the absence of recordable activity in collected points having bipolar voltage amplitude ≤ 0.03 mV (baseline noise in the Biosense system). SNO zones were the result of the coherent mapping algorithm, exhibit absence of conduction velocity vectors on coherent map, and appear as brown on the 3D maps (Figure 1A).^15^ For optimal performance, the following were likewise adjusted.

- Scar settings were defined with a voltage cutoff of less than 0.05 and less than 0.5 mV used to define scar and low voltage zones, respectively.^16^
- Signals that were identified as double‐potential and fractionated were tagged.
- The interpolation threshold of the color fill‐in of the reconstruction was set at 5% during map acquisition to permit a relatively uniform density of mapping points.
- Anatomical structures were marked and cut before coherent map calculation.

All the maps were interpreted by two operators (JJBV and PTL), if there were disagreements in the map interpretation, a third operator (YJL) was consulted. The standard LAT map was evaluated first with adjustments made with results of entrainment mapping and the use of the extended early‐meets‐late (EEML) feature of the software. This was followed by calculation of the coherent map. The ablation strategy was performed based on the interpretation of the coherent map.

#### 2.3.2 Standard activation and propagation

The details of standard EAM has been described previously.^7^ In brief, a sharp and stable signal from a decapolar catheter inserted into the coronary sinus was used to provide the timing reference signal during the mapping procedure.^11^ After completing an intact right atrial (RA) and/or LA geometry reconstruction, depending on suspected chamber site of origin based on surface ECG P wave morphology,^12^, ^17^ a multielectrode mapping catheter was selected as the roving catheter that was used to collect the LAT (relative to the reference signal) and voltages. The signal from the roving catheter was used to build a sequential map. Total activation time was defined as the time interval from the earliest to the latest activation point in the atrium.^7^ The extended early‐meets‐late feature of the software was turned on during map review to aid map interpretation. This feature highlights areas of potential conduction block thereby providing a basis for a better interpretation of the LAT and propagation map.^18^

#### 2.3.3 Coherent mapping

A coherent map was reconstructed online using the investigational module of CARTO 3 system (Coherent Mapping) with the same points collected during activation mapping and with the addition of color and conduction velocity vectors for the representation of the electric wave propagation over the heart chamber. Coherent mapping is a CARTO 3 system feature that improves the representation of the electric wave propagation over the atria by means of coloring and direction vectors, focusing on the displayed path of cyclic arrhythmia propagation. The reconstruction includes the detection of areas with probable conduction barriers (SNO zones). This algorithm was designed based on the electrophysiological conditions of typical flutter, atypical flutter, and focal AT wherein conduction velocity is continuous and there are no sharp turns of wave velocity, except in areas with slow conduction or blocks with tissue properties as defined in the literature. This algorithm uses the LAT values of all the points in a map and performs an iterative calculation for a global best‐fit solution in these arrhythmias, analogous to calculating a regression line to show how scattered points indicate a global trend. The main conditions used by the algorithm are:

- low (bipolar voltage) potential points such as scar,
- double‐potential points, and
- dense map of the chamber with uniform distribution of points.

Compared with standard LAT coloring which presents a local solution and where every point has an individual contribution to the map coloring, coherent coloring presents a global best‐fit solution based on all the map points.

SNO zones which represent probable conduction barriers with slow conduction or blocks were defined by the absence of recordable activity, bipolar voltage amplitude ≤ 0.03 mV (baseline noise in the Biosense system) and absence of conduction velocity vectors on coherent map and appear as brown on the 3D maps. SNO sites were identified by calculating the coherent map and conduction velocity vectors in relation to the surrounding electrograms and were displayed as brown color on top of their coherent colors.^5^ Dynamic conduction velocity vectors were projected, and conduction velocity was adjusted to highlight slow conducting areas represented by thicker conduction velocity vectors relative to the conduction velocity of the mapped chamber on the electroanatomic shells.

The conduction velocity (CV) threshold was adjusted individually when reviewing each map with the goal of identifying area/s where conduction velocity slows relative to the entire map, which would suggest a potential deceleration zone and an isthmus site. There was no single threshold used for all maps.

#### 2.3.4 Coherent map interpretation and identification of critical isthmus sites

Maps were reviewed and evaluated for the presence of focal activation or macroreentry. In cases of macroreentry, critical isthmus sites, defined as the narrowest region of orthodromic conduction in the isthmus bounded on both sides by SNO regions (brown areas on the coherent map) due to functional or permanent block as determined from the coherent map or by block on one side and an anatomic edge on the other.^19^

The critical isthmus was identified and measured by adjusting the displayed scar threshold setting between 0.03 and 0.05 mV, only including points above the background noise setting. Figure 1 illustrates the effect of changing the scar threshold on both the coherent and LAT maps with a more apparent change noted on the coherent map recalculated after each scar threshold change. The design line tool was used to identify the different circuits in ATs with multiple wave propagations. Participation in the circuit was confirmed by entrainment mapping and verified by successful consecutive ablation.^1^, ^20^ Entrainment was attempted on all isthmus‐dependent re‐entrant ATs but was only possible in 17 (77.3%) due to termination of the tachycardia during pacing attempt in 2 (9.1%) and changing cycle length in 3 (13.6%). Bystander circuits were identified as circuits with activation traveling away from an isthmus and splitting around regions of SNO zones or anatomic structures. Retrospective offline measurement of conduction velocity was performed by dividing the distance between successively activated points by the time difference of the respective LAT of the same points except in areas of collision of different wavefronts. Isthmus sites were defined by: concealed entrainment (PPI < 20 ms), CV less than 0.3 m/s,^21^, ^22^ and local fractionated electrograms at sites bordered by SNO zones or anatomic structures.

### 2.4 Catheter ablation

**Figure 2:**
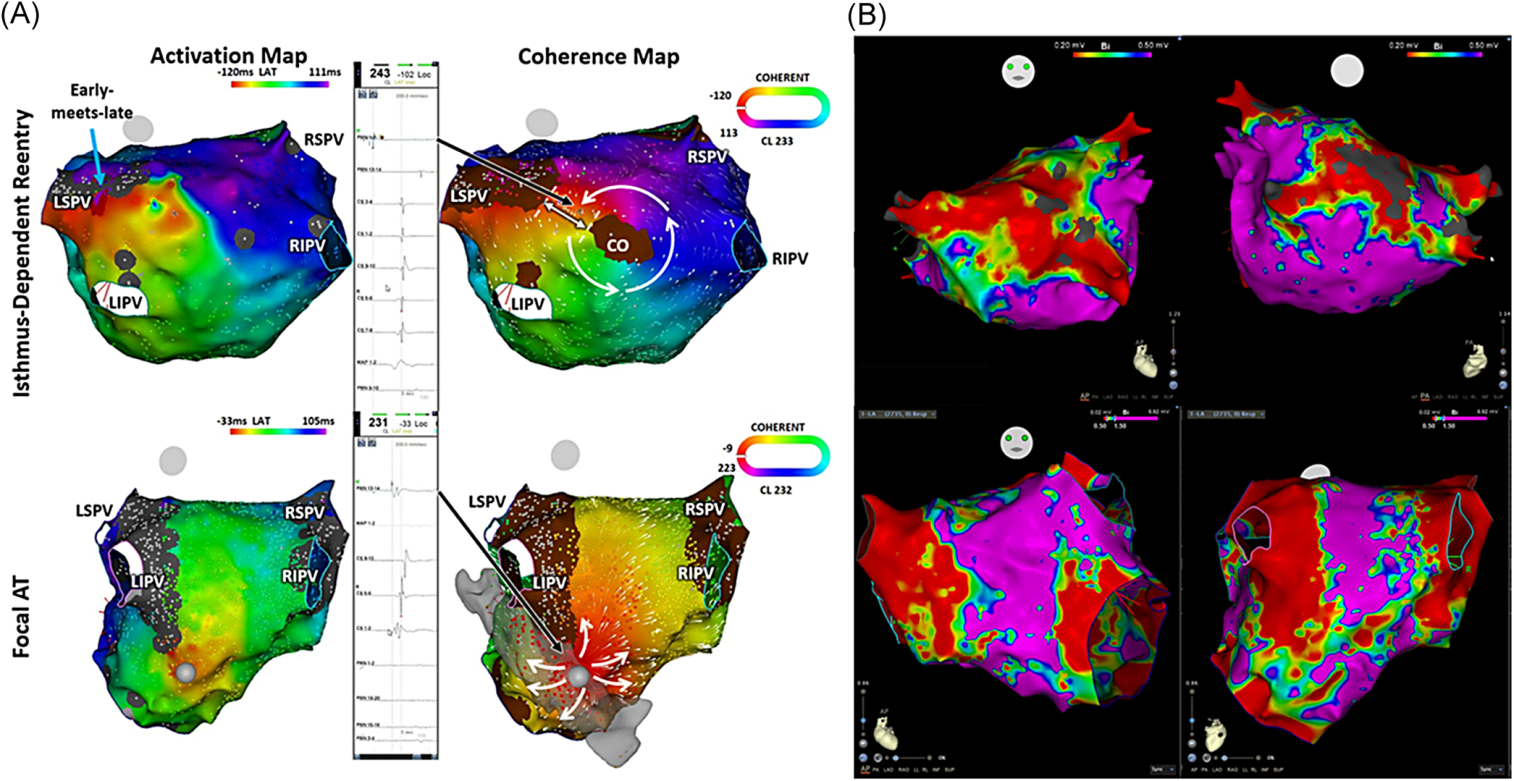
A, Sample cases 1 and 2. Examples of critical sites: isthmus‐dependent re‐entry (IDR; upper panel) and focal AT (lower panel) on activation (left panel) and coherent map (right panel). IDR on coherent map (upper right panel) at the left atrial posterior wall with the circuit rotating around a nonconducting central obstacle (CO) represented by a brown SNO site with identifiable slow conduction (fractionated electrogram, upper middle panel) at narrow isthmus site (white double‐ended arrow), bordered by the nonconducting CO and another SNO site, that was not seen on standard activation mapping (upper left panel). Focal activation with the radial spread at breakout site (earliest activation site) identified by both activation and coherent maps. B, Corresponding voltage map of sample case 1 (isthmus‐dependent AT, top panel) and case 2 (focal AT, bottom panel) (Figure 2A above) acquired during sinus rhythm. AT, atrial tachycardia; LIPV, left inferior pulmonary vein; LSPV, left superior pulmonary vein; RIPV, right inferior pulmonary vein; RSPV, right superior pulmonary vein; SNO, slow or nonconducting

A planned ablation lesion set was defined based on the studied coherent map targeting the earliest activation sites for focal AT and slow conduction isthmus (CI) of re‐entrant/isthmus‐dependent AT. For isthmus‐dependent ATs, linear ablation lesions were targeted at the slow CI defined by the narrowest isthmus bordered by SNO zones and/or anatomical barriers with thicker conduction velocity vectors relative to the entire map. Sample case 1 (Figure 2A, right upper panel) demonstrates the narrowest isthmus site (white double‐ended arrow) bordered by a nonconducting central obstacle (CO) and an SNO site where ablation was applied and terminated the tachycardia. Radiofrequency ablation was performed under power control mode with a maximal power of 25 to 35 W using an open‐irrigated tip ablation catheter and delivered for at least 120 seconds at the origin or breakout site of focal AT. For isthmus‐dependent ATs, the ablation lesions were performed continuously while repositioning the catheter tip every 40 seconds between nonconducting sites that allowed crossing the critical isthmus.^8^, ^9^, ^12^ The common isthmus was not always targeted and depended on the preference of the operator such as the decision to ablate the anatomical isthmus or cavotricuspid isthmus for peritricuspid re‐entrant tachycardia. Ablation was considered successful if the tachycardia terminated or changed to a different activation pattern with an associated change in cycle length and negative inducibility of clinical AT by programmed extra stimuli from the CS catheter with intravenous isoproterenol (1‐4 μg/min) infused to achieve at least a 20% heart rate increment.^13^, ^14^, ^23^ If tachycardia transitioned to another AT, the operators were encouraged to remap the atrial chamber of interest.

### 2.5 Follow‐up

Patients were followed‐up at our cardiology clinic or with the referring physicians initially at 2 weeks after discharge, then every 1 to 3 months thereafter. Patients with concurrent AF were prescribed with antiarrhythmic drugs for 2 months to prevent early recurrences. On each follow‐up, 12L ECG was performed and if patients complained of symptoms suggestive of arrhythmia, a 24‐hour Holter monitoring and/or cardiac event recording for 1 week was performed. Atrial tachyarrhythmia recurrence was defined as an episode lasting more than 1 minute and confirmed by an ECG occurring after 3 months of the blanking period. If more than one episode of recurrent symptomatic atrial arrhythmia was documented, the patients were encouraged to receive a second ablation procedure or were prescribed antiarrhythmic drugs to control recurrence.^7^

### 2.6 Statistical methods

Parametric data are reported as mean ± SD. A *χ* ^2^ with a Fisher's exact test was used for categorical data. The student independent *t* test with Levene's test for homogeneity was used for continuous data. Correlation of numeric variables was assessed with the Pearson's correlation coefficient with Phi and Cramer's V tests for association. A value of *P* < .05 was considered statistically significant.

All mapped ATs were examined and scored independently by five observers. On a separate occasion two of the observers repeated the assessments of the same LAT and coherent maps in the absence of information from the initial observations. The chance corrected and weighted kappa statistics for observer agreement for interobserver and the linear correlation between the measurements (Spearman's coefficient of correlation) and their reliability through intraclass correlation coefficient (ICC), the Cronbach's *α* coefficient and the limits of agreement proposed by Bland and Altman were evaluated.

All analyses were conducted with SPSS Statistics for Windows (version 17.0; SSS Inc, Chicago, IL).

## 3 RESULTS

### 3.1 Patient characteristics

Twenty patients (15 males, mean age: 58.2 ± 10.3 years old) met the inclusion criteria and were included in the study; nine with history of cardiac surgery (five for valvular heart disease, two with concurrent coronary arterial bypass graft [CABG], and another two with concurrent MAZE procedure), two with the previous repair of congenital atrial septal defect, two with previous CABG, 16 with history of previous atrial ablation and one that developed AT after AF ablation. Hypertension was present in 35%, diabetes in 15%, heart failure in 5%, coronary artery disease in 30%, hyperlipidemia in 20%, valvular heart disease in 25%, previous atrial arrhythmia ablation in 80% and prior cardiac surgery in 45%. The mean left ventricular ejection fraction (LVEF) was preserved (mean: 59.6% ± 9.4%) with only two patients with LVEF less than 55%. The mean left atrial diameter (LAD) was 45.7 ± 9.8 mm with only five patients with LAD of less than 40 mm. Tables 1 and S1 provides a summary of the clinical cases.

**Table 1:** Clinical characteristics

Variables	Patient (n = 20)
Age, y	58.2 ± 10.3
Sex, male	15 (75%)
Hypertension	7 (35%)
Diabetes	3 (15%)
Congestive heart failure	1 (5%)
Coronary artery disease	6 (30%)
Hyperlipidemia	4 (20%)
Stroke/CVA	2 (10%)
Valvular heart disease	5 (25%)
Atrial fibrillation	19 (95%)
Previous atrial ablation	16 (80%)
Mean number of ablations	2.3 ± 3.0
Surgery for structural heart disease (VHD/CHD)	9 (45%)
Smoking	3 (15%)
Alcohol	4 (20%)
LVEF, %	59.6 ± 9.4
LAD, mm	45.7 ± 9.6

Abbreviations: CHD, congenital heart disease; CVA, cerebrovascular accident; LAD, left atrial diameter; LVEF, left ventricular ejection fraction; VHD, valvular heart disease.

## 4 MAPPING DETAILS

### 4.1 Electrophysiological characteristics of the ATs

#### 4.1.1 Baseline rhythm and inducibility

Baseline presentation was atypical LA flutter in 11, sinus rhythm in 5, typical peritricuspid atrial flutter in 3, AF in 3, LA AT in 2, and scar‐related RA flutter in 2. AT induction was performed in the five patients who presented in sinus rhythm while three patients who presented in AF underwent PVI before AT mapping and ablation.

#### 4.1.2 Mapping site details

The mean mapping time was 29.5 ± 24.0 minutes which garnered a mean of 2083.5 ± 1021.0 points per chamber mapped. The mean bipolar voltage per chamber is 0.67 ± 0.67 mV (0.56 ± 0.66 on the LA and 0.92 ± 0.67 mV on the RA; *P* = .683) and SNO areas defined by coherent mapping occupied 14.0% ± 8.6% (14.31 ± 8.16 on the LA and 13.32% ± 10.05% on the RA; *P* = .325) of the chamber mapped (Table S1) after adjusting the CV threshold setting to the optimum ability to identify a potential isthmus.

#### 4.1.3 Map results based on the coherent map

Out of 35 identified ATs (averaged 1.75 per patient), only 26 (4 focal and 22 isthmus‐dependent) were mappable and were included in the analysis comparing LAT and coherent mapping. Out of the 26 mapped ATs, 4 were focal, and 22 were isthmus‐dependent. The majority were from the LA (18/22, 69.23%), eight were perimitral (one focal perimitral, one focal CS, and six macroreentrant), six from the LA roof, two from the left pulmonary veins, one from the right pulmonary vein, and one from the LA free wall. Eight (8/26, 30.80%) of the ATs were mapped from the right atrium, six were peritricuspid isthmus‐dependent flutters and two were from previous surgical scars.

#### 4.1.4 Map results based on standard activation maps

**Figure 3:**
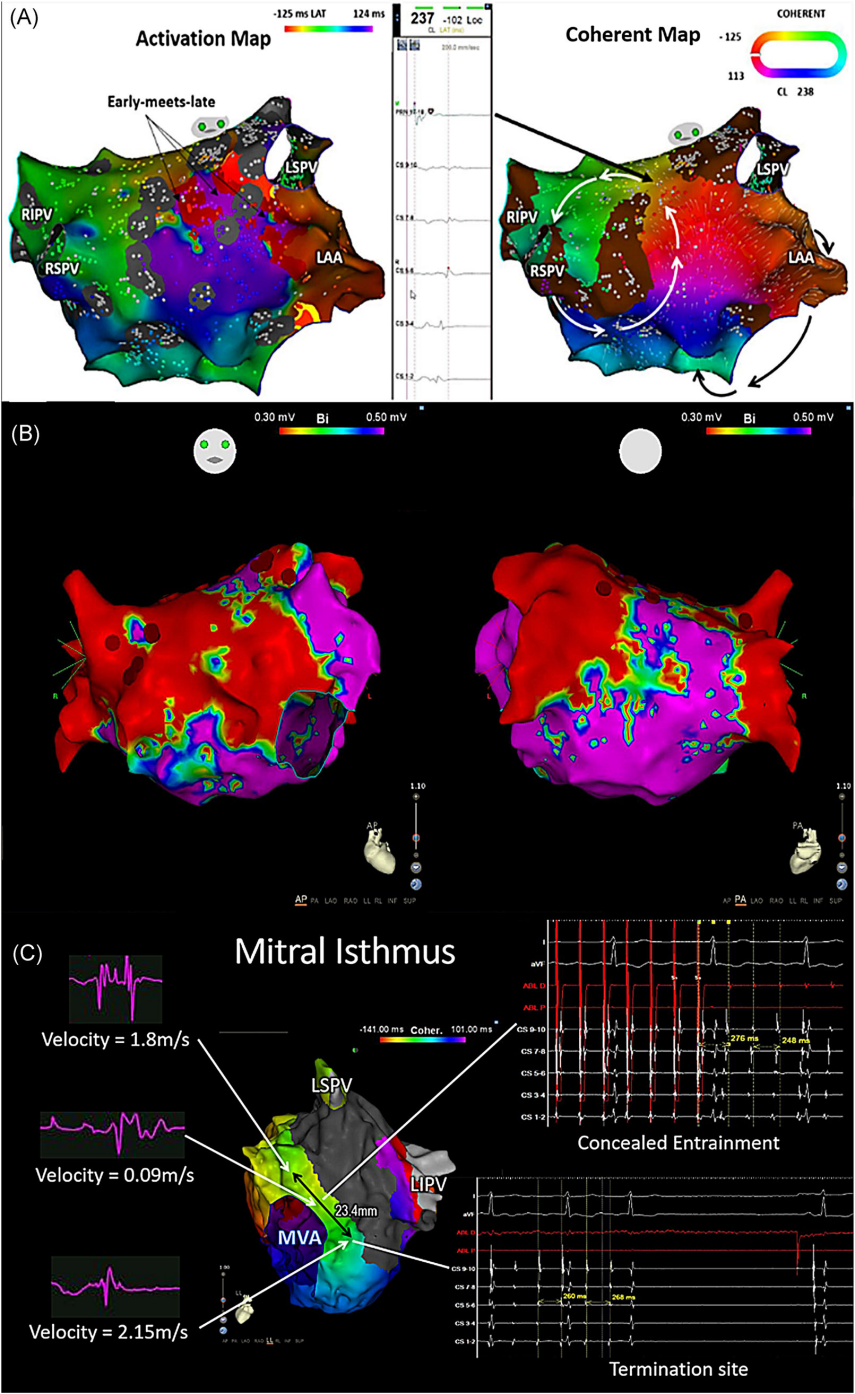
Sample case 3 comparisons of the standard activation map and coherent map. A, Top left panel: activation map showing early‐meets‐late at the left atrial (LA) roof and base of the LAA. Top middle panel: Fractionated electrogram at the critical isthmus identified by coherent map at the LA roof. Top right panel: coherent map showing multiple loops of the AT with the narrowest width located at the LA roof and a common isthmus at the LA anterior wall shared by a roof macroreentrant circuit and another circuit rotating around the mitral annulus. B, Corresponding voltage map of sample case 3 (Figure 3A above) acquired during sinus rhythm. C, Bottom panel: critical isthmus site bordered by anatomical and functional nonconducting areas showed slow conduction, concealed entrainment and was verified by termination with ablation. AT, atrial tachycardia; LA: left atrium; LAA, left atrial appendage; LAT, local activation time; LIPV, left inferior pulmonary vein; LSPV, left superior pulmonary vein; RA, right atrium; RIPV, right inferior pulmonary vein; RSPV, right superior pulmonary vein

After review of the coherent map and conventional activation map, 22 of the 26 (84.6%) AT cases were diagnosed as an isthmus‐dependent circuit. The rest of the four ATs exhibited a centrifugal activation (two from the left superior pulmonary vein, one from the posterior mitral annulus, and one from the coronary sinus; Figure 2, Videos S1 and S2). Conduction velocity vectors demonstrated the direction of wave propagation and splitting along nonconducting sites and anatomical barriers (Figures 2 and 3, Videos S1‐S3). Out of the 26 ATs, only 18 (69.2%; *P* = .010) were interpretable with the LAT map, 14 (53.8%; *P* = .039) were isthmus‐dependent and 4 (15.38%; *P* = .99) exhibited centrifugal activation pattern on LAT map.

The LAT maps were extended to 80% to 100% during map review, however, there was no identifiable potential isthmus in 30.8% of the LAT maps.

Figure 1 (Video S3) demonstrates an example of isthmus‐dependent re‐entrant AT (upper panel) and focal AT (lower panel) with a comparison of the LAT map and coherent map. There is no difference in the location of the identified focal origin for the focal AT between the LAT map and the coherent map, however, in the isthmus‐dependent re‐entrant AT, the coherent map clearly showed the conduction velocity vectors and wavefront propagation coursing around a nonconducting CO (Table 2).

**Table 2:** Interpretation of coherence map and LAT mapping

	LAT map (n = 26)	Coherence map (n = 26)	*P* value
Interpretable	18 (69.2%)	25 (96%)	**.010**
Focal	4 (100.0%)	4 (100%)	.99
Isthmus‐dependent re‐entry	14 (53.8%)	21 (80.8%)	**.039**
Isthmus dimension, mm	43.0 ± 23.9	19.8 ± 10.5	**<.001**
Critical site location			.168
Mitral/CS	6 (23%)	8 (31%)	.532
LA roof/free wall	5 (19%)	7 (27%)	.510
PV	1 (4%)	3 (12%)	.298
CTI	4 (15%)	6 (23%)	.482
RA scar/septum	2 (8%)	1 (4%)	.552

*Note*: Values are mean ± SD or n (%). Values <0.05 were considered as significant and are highlighted in bold.

Abbreviations: CS, coronary sinus; CTI, cavotricuspid isthmus; LA, left atrium; LAT, local activation time; PV, pulmonary vein; RA, right atrium.

### 4.2 Comparison of standard EA mapping and coherent mapping

Coherent mapping was able to identify critical isthmus/focal origin sites where ablation terminated the tachycardia in 96.2% compared with conventional mapping (69.2%; *P* = .010). Significantly more CI (mean/chamber 2.0 ± 1.1 vs 1.0 ± 0.7; *P* < .001) with narrower width (19.8 ± 10.5 vs 43.0 ± 23.9 mm; *P* < .001) were identified by coherent mapping than standard activation mapping (Table 2). Single circuit ATs corresponded with single CI (*P* < .001) while multi‐circuit ATs corresponded with ≥3 CI sites (70%; *P* = .003). Figure 3A demonstrates a case of multi‐circuit AT with a comparison of the standard LAT map and coherent map. The LAT map demonstrates the early‐meets‐late at the anterior LA roof and base of the LA appendage. The coherent map demonstrates multiple loops on the same case, with the narrowest isthmus identified at the LA roof and another common isthmus shared by the LA roof circuit and perimitral circuit at the LA anterior free wall. Slowing of conduction at the isthmus sites is demonstrated by thicker conduction velocity vectors (Video S3). Figure 3B demonstrates the critical isthmus site of a mitral flutter bordered by anatomical and functional nonconducting areas with slow conduction, concealed entrainment, and verified by termination with ablation. Coherent mapping significantly identified more ATs with conduction isthmus ≥ 3 compared with LAT mapping which was not able to identify conduction isthmus ≥ 3 (Table 3).

**Table 3:** Characteristics of ATs identified by LAT map and coherence map

	LAT map	Coherence map	*P* value
Number of circuits/chamber	1.0 ± 0.76	1.45 ± 0.51	**.024**
Single circuit	10 (62.5%)	12 (54.5%)	.546
Double circuit	6 (37.5%)	10 (45.5%)	.210
Conduction isthmus/AT	0.95 ± 0.67	2.0 ± 1.07	**<.001**
1	10 (62.5%)	12 (54.5%)	.443
2	4 (25.0%)	4 (18.2%)	.942
≥3	0 (0.0%)	8 (36.4%)	**.002**
Isthmus dimension, mm	30.01 ± 14.67	16.83 ± 5.34	**<.001**

*Note*: Values <0.05 were considered as significant and are highlighted in bold.

Abbreviations: AT, atrial tachycardia; LAT, local activation time. Values <0.05 were considered as significant and are highlighted in bold.

A CI number ≥3, 2 and 1 corresponded to 7 (70%), 3 (30%) and 0 (0%) of multi‐circuit AT (Table 4) and to 3 (60%) multiple, 1 (25%) double and 9 (69%) single clinical ATs, respectively (Table 5).

**Table 4:** Correlation of number of circuits and conduction isthmus

Conduction isthmus	Multi‐circuit AT (n = 10)	Single circuit AT (n = 12)	*P* value
1	0 (0.0%)	10 (83.3%)	**<.001**
2	3 (30.0%)	1 (8.3%)	.190
≥3	7 (70.0%)	1 (8.3%)	**.003**

*Note*: Values <0.05 were considered as significant and are highlighted in bold.

Abbreviation: AT, atrial tachycardia.

**Table 5:** Correlation of number of clinical AT and conduction isthmus

Conduction isthmus	Single AT (n = 13)	Two AT (n = 4)	Multiple AT (n = 5)	*P* value
1	9 (69.2%)	0 (0.0%)	1 (20.0%)	**.022**
2	2 (15.4%)	1 (25.0%)	1 (20.0%)	.903
≥3	2 (15.4%)	3 (75.0%)	3 (60.0%)	**.044**

*Note*: Values <0.05 were considered as significant and are highlighted in bold.

Abbreviation: AT, atrial tachycardia.

Figure S1 shows that the isthmus velocity was slower than the entrance or the exit of the isthmus on the coherent map (*P* < .01) with the calculated CV of 0.57 ± 0.20 m/s and mean bipolar voltage of 0.43 ± 0.16 mV at the isthmus site.

### 4.3 Outcomes of ablation

Ablation at the identified critical conduction channels based on coherent mapping terminated the tachycardia to sinus rhythm in 18 ATs (69.2%) and changed the rhythm to another AT with different activation wavefront and cycle lengths in seven (26.9%). In four cases (two multiple AT from the LA and two scar‐related AT from the RA) with multiple circuits identified on coherent mapping, linear ablation at the narrowest isthmus of the first circuit caused a change in tachycardia cycle length and atrial activation sequence. Remapping showed a change in the electric wave propagation to the remaining circuit. Linear ablation directed at the remaining narrowest isthmus was performed and successfully terminated these ATs to sinus rhythm. Ablation at focal origin and CI sites successfully terminated the tachycardia with negative inducibility of the same AT in 25 (96.2%; Table 2).

### 4.4 Long‐term follow‐up

On long‐term follow‐up (mean: 409.9 ± 137.3 days), 25% of the patients were documented to have a recurrence (three with atrial flutter and two with AF) with a mean time to recurrence of 379 ± 148 days. The majority (15/20, 75%) of the patients were in sinus rhythm on a follow‐up including one patient with initially failed ablation procedure, with 35% (four in the recurrence and three in the no recurrence groups) maintained on antiarrhythmic drugs. Patients with recurrence were significantly more likely to be maintained on antiarrhythmic drugs on long‐term follow‐up (logrank *P* = .0253).

### 4.5 Inter‐ and intraobserver variability

The chance corrected and weighted kappa statistics for observer agreement, both for interobserver and intraobserver variability demonstrated satisfactory repeatability for both coherent and LAT maps. The overall intraobserver mean weighted kappa was *κ *= 1 (*P* < .001) for coherent maps and *κ* 0.74 (*P* = .01) for the LAT maps. The overall interobserver reliability was likewise above the standard limits with Cronbach's *α* = 1 [ICCR: 1 (1‐1)] for coherent maps and Cronbach's *α* .93 [ICCR: 0.93 (0.81‐0.98)] for LAT maps.

## 5 DISCUSSION

### 5.1 Main findings

First, the addition of conduction velocity vectors as calculated by the coherent mapping global best‐fit solution using points collected from contiguous sites improved identification of nonconducting areas bordering critical isthmus sites and complex atrial electric wave propagation that facilitated ablation. Second, common isthmuses from multiple atrial circuits and dual‐loop circuits and bystander circuits that terminate in nonconducting sites or anatomical barriers were easily recognizable. Third, patients who presented with multiple ATs were demonstrated to have at least three identifiable CI sites using coherent mapping compared with the standard LAT map.

### 5.2 Comparison with previous studies

Our study confirms the outcome of the recent study by Anter et al^5^ on the utility of vectors generated from adjacent points in the identification of the mechanisms and critical site location of complex scar‐related ATs. In addition to this, we demonstrate improved recognition of critical isthmus sites, multiple circuits, dual‐loop circuits, and bystander circuits through activation voltage thresholding to project nonconducting areas. Previously, Huang et al^24^ demonstrated that conduction velocities within isthmus paths were significantly slower than outside paths with the protected isthmus bordered by low voltage zones characterized by normalized peak negative voltage zones. The use of coherent mapping simplified the identification of slow conducting areas relative to the surrounding areas by projecting thicker conduction velocity vectors. These conduction barrier/s are anatomical or functional barriers, and the connection of the CIs through these barriers is able to treat both single and multiple loop AFL with comparable efficacy to the anatomic approach (eg, roof line or mitral line for roof flutter or mitral flutter, respectively).

### 5.3 Diagnosis and ablation of scar re‐entrant AT

A change in cycle length and activation pattern during ablation occurred in seven of the ATs included in our study likely due to the presence of codominant circuits. Although, the most intuitive ablation strategy is to target the common isthmus as ablation of one loop may produce a sudden transformation to a new re‐entrant tachycardia formed by the remaining loop that requires ablation at a second isthmus.^25^ However, sometimes radiofrequency ablation of two distinct isthmuses may be a better option than transection of the common isthmus due to location or a wider diameter of the common isthmus than separate multiple isthmuses.^26^ Seiler et al^26^ attributed the sudden change in cycle length during ablation to a shorter revolution time (dominant loop) of the loop targeted with radiofrequency ablation than the second loop. If both loops have the same revolution time (codominant loops), or if the loop with a longer revolution time is first targeted with ablation, there was no change in cycle length subsequent to the disconnection of the first isthmus.^26^ Previously, dual‐loop circuits should only be systematically anticipated in patients with intra‐atrial re‐entrant tachycardia late after open‐heart surgery and should be suspected in case of tachycardia change during radiofrequency ablation.^25^ Our study showed that the coherent mapping module can clearly identify multiple circuits through the projected conduction velocity vectors and SNO sites. However, the complexity of the AT mechanism and circuits can still cause a change in the propagation and transfer to another existing circuit after the ablation of the first circuit. The identification of all the loops is critical to the ablation strategy which targets the common isthmus when at least one nonanatomic loop is involved and is reported by Takigawa et al^27^ to achieve acute success in terminating the tachycardia in 80%. It is therefore essential to determine whether one loop is a “functional” circuit that will keep rotating once the first loop is abolished by radiofrequency ablation of the other loop, or whether it is an “innocent” bystander that will not require additional treatment.^26^ Recently, Takigawa et al^27^ reported that when dual‐loop ATs included a nonanatomic loop, the common isthmus was generally short, and the ablation was successful. When the arrhythmia combined two nonanatomic loops, a complete anatomic linear ablation was usually not needed and accounted for the better outcome observed in their study. On the contrary, when the dual‐loop AT included two anatomical multifocal AT circuits, a complete anatomic isthmus block was required.^27^ Verma et al,^28^ demonstrated improved outcomes after ablation of all potential isthmuses in postoperative RA incisional scar and flutter. The continuation of the propagation of impulses in the remaining AT circuit in multiple loop ATs in our study may have accounted for the longer time needed for radiofrequency ablation. This implies that early recognition and vigilance to the detection of cycle length and activation pattern change, especially in multiple loop ATs that are easily recognized in the coherent map, is essential to potentially improve outcomes of ablation.

### 5.4 Diagnosis and ablation of focal type of AT

This study showed similar sites of origins in the four cases of focal ATs identified by both the standard activation map and the recalculated coherent map (Figure 2, Video S2) with centrifugal activation patterns from the site of origin. In their study, Anter et al^5^ was able to identify localized re‐entry with the coherent map implying better recognition of mechanisms of the arrhythmia, however, all of the four cases included in our study exhibited centrifugal activation pattern on both the coherent map and the standard activation map suggesting focal mechanism and confirmed by successful ablation at the focal source. Similar to our previous findings,^16^ this study showed that focal ATs tended to originate from areas of low voltage (0.39 ± 0.21 mV).

### 5.5 Clinical implication

Entrainment mapping combined with 3D EAM allows delineation of complex re‐entry circuits and critical isthmuses as targets for ablation,^29^ however, both techniques have their own inherent limitations. The addition of conduction velocity vectors calculated using the coherent mapping global best fit and based on contiguous points, bypassed most of the limitations imposed by conventional mapping strategies and improved the correct identification of nonconducting tissues and critical isthmus sites that supported re‐entry in complex ATs compared with conventional EAM. Complex electric wave propagation and splitting of waves in anatomical obstacles and nonconducting sites that resulted in bystander circuits were easily identified. Common isthmuses formed by multiple circuits were visualized and the vigilant recognition of changing cycle length and activation sequence in multiple loop circuits could potentially improve the ablation outcome.

## 6 LIMITATIONS

First, this study is performed using a new investigational mapping module that needed a learning curve for accurate interpretation. Second, this study is limited by the small sample size of complex AT based on the history of prior atrial interventions. This small sample size additionally limits the identification of all mechanisms reported in previous studies of coherent mapping. Third, ATs with cycle length instability possibly representing complex AT were excluded from the study. Fourth, all maps were acquired during tachycardia to facilitate the identification of functional nonconduction zones.

## 7 CONCLUSIONS

The coherent mapping facilitates recognition of critical isthmus sites through identification of visually available display of nonconducting (SNO) zones and facilitate recognition of slow conduction areas through thicker conduction velocity vectors relative to the surrounding areas. Catheter ablation targeting the identified CIs successfully eliminated the scar‐related atrial flutter of the left and right atrium.

## CONFLICT OF INTERESTS

The authors declare that there are no conflict of interests.

## Supporting information

Supporting information

Supporting information

Supporting information

Supporting information

Supporting information

Supporting information
