# Supplementary material for: Identification of critical isthmus using coherent mapping in patients with scar‐related atrial tachycardia
Source: J Cardiovasc Electrophysiol. 2020 Apr 6;31(6):1436–47. doi: 10.1111/jce.14457 (PMC7383970; doi:10.1111/jce.14457)
Supplement: Supplementary file 2 — Supporting information [file JCE-31-1436-s002.docx]

**Supplemental Table 1: Electrophysiologic Properties of mapped ATs**

|  | **Focal (n=4)** | **Macroreentry (n=22)** | **P value** |
| --- | --- | --- | --- |
| Location |  |  |  |
| Left atrium | 4 (100%) | 14 (63.6%) |  |
| LA roof | 0 (0%) | 6 (42.9%) | 0.929 |
| Pulmonary Vein | 2 (50%) | 1 (7.1%) | 0.580 |
| Peri-Mitral/CS | 2 (50%) | 6 (42.9%) | 0.747 |
| Free wall | 0 (0%) | 1 (7.1%) | 0.679 |
| Right atrium | 0 (0%) | 8 (36.4%) |  |
| Peri-tricuspid isthmus | 0 (0%) | 6 (75%) | 0.001 |
| RA scar | 0 (0%) | 2 (25%) | 0.029 |
| AT cycle length, ms | 238±30 | 252±35 | 0.432 |
| Total RFA Pulses | 14.25±11.62 | 21.9±19.88 | 0.467 |
| Total RFA time, sec | 570±464 | 905±799 | 0.428 |
| Total procedure time, min | 40.25±19.83 | 75.55±51.49 | 0.195 |
| Total fluoroscopy time, min | 27.58±15.40 | 46.27±36.68 | 0.332 |
