# Supplementary material for: Identification of critical isthmus using coherent mapping in patients with scar‐related atrial tachycardia
Source: J Cardiovasc Electrophysiol. 2020 Apr 6;31(6):1436–47. doi: 10.1111/jce.14457 (PMC7383970; doi:10.1111/jce.14457)
Supplement: Supplementary file 6 — Supporting information [file JCE-31-1436-s006.docx]

**Supplemental Video 1**

**Sample Case 1 comparing standard Propagation Map (left panel) and Coherent Map (right panel) of an isthmus-dependent macroreentrant circuit at the left atrial posterior wall.** The Propagation map shows counterclockwise circuit at the posterior left pulmonary vein area with slowing of conduction at the junction of the posterior left pulmonary vein and left atrial roof area. The Coherent map shows a counterclockwise circuit rotating around a central obstacle (brown area at the center of the circuit) with thicker dynamic conduction velocity vectors at the isthmus between the posterior left superior pulmonary vein and left atrial roof. The Coherent map demonstrates a narrower conduction isthmus bordered by the central obstacle and anatomic non-conduction site (left superior pulmonary vein).

**Supplemental Video 2**

**Sample Case 2 comparing standard Propagation Map (left panel) and Coherent Map (right panel) of a focal AT localized at the low left atrial wall adjacent to the posterior mitral/coronary sinus area.** The propagation map demonstrates color atrial wave propagation away from the central break-out site while the Coherent map shows dynamic conduction velocity vectors moving away from the central break-out site.

**Supplemental Video 3**

**Sample Case 3 comparing standard Propagation Map (left panel) and Coherent Map (right panel) of an isthmus-dependent macroreentrant AT with multiple conduction isthmus at the LA roof and anterior free wall.** The propagation map demonstrates early-meets-late at the LA roof and LA appendage with a fast counter-clockwise atrial wave propagation at the LA roof and slow clockwise atrial wave propagation at the LA anterior free wall and another fast-clockwise atrial wave propagation below the LA appendage and slow conducting atrial wavefront at the anterior mitral annulus. The Coherent map shows a figure-of-8 macroreentrant AT with multiple conduction isthmus, 1 loop with a narrow isthmus at the LA roof and another loop at the peri-mitral area with a common isthmus at the LA anterior free wall demonstrating relative slow conduction with thicker conduction velocity vectors at the isthmus areas.

**Supplemental Figure 1**

The conduction velocity of the isthmus at the entrance, critical isthmus, and exit. *p<0.01.
